# Cardiac osteosarcoma: a case report and literature review

**DOI:** 10.3389/fcvm.2023.1215389

**Published:** 2023-07-10

**Authors:** Dae-Hwan Bae, Sangshin Park, Min Kim, Sangmin Kim, Woong Gil Choi, Jang-Whan Bae, Kyung-Kuk Hwang, Dong-Woon Kim, Myeong-Chan Cho, Ju-Hee Lee

**Affiliations:** ^1^ Department of Cardiology, Chungbuk National University Hospital, Cheongju, Republic of Korea; ^2^ Department of Cardiology, Chungbuk National University College of Medicine, Cheongju, Republic of Korea

**Keywords:** cardiac osteosarcoma, cardiac tumor, chemotherapy, echocardiography, surgery

## Abstract

**Background:**

Primary cardiac tumors are rare, and malignant primary cardiac tumors are even rarer. Cardiac osteosarcoma is a very rare type of malignant primary cardiac tumor with limited reported cases. We present a case report of cardiac osteosarcoma and review its characteristics and the related literature.

**Case summary:**

A 44-year-old female patient without a specific medical history presented with intermittent dyspnea that started 1 month prior to presentation. A heterogeneous mass was observed in the left atrium on echocardiography and a large mass was observed in the left atrium on computed tomography. Surgery was performed under the suspicion of atypical cardiac myxoma, and the tumor was successfully removed. However, postoperative histopathological examination revealed cardiac osteosarcoma. The patient underwent chemotherapy and has been well maintained without recurrence for 10 years.

**Conclusion:**

We present a case report of the echocardiographic features and treatment strategies for cardiac osteosarcoma, an extremely rare cardiac tumor. Multimodal imaging can be helpful; however, a histological diagnosis through surgical resection is essential. Appropriate treatment and follow-up based on histological findings are necessary.

## 1. Introduction

Cardiac tumors are classified as primary or metastatic tumors. Metastatic cardiac tumors are the most common type and are approximately 30 times more common than primary cardiac tumors (1). Benign primary cardiac tumors account for approximately 80% of all cases, with myxomas being the most common, followed by lipomas, papillary fibroelastomas, and rhabdomyomas. Malignant primary cardiac tumors are rare and predominantly comprise various forms of sarcoma (2). Cardiac sarcomas are usually poorly differentiated, which often makes their precise histological classification difficult. The most common sarcomas are tumors of vascular origin, particularly angiosarcomas (3, 4). However, rare cases of bony, neurogenic, and soft tissue sarcomas have reportedly arisen from the cardiac tissue. Primary cardiac osteosarcoma is extremely rare and mainly occurs in the left atrium (5). Herein, we present a case of primary cardiac osteosarcoma diagnosed by pathological examination of a surgical specimen.

## 2. Case description

A 44-year-old woman visited our cardiology outpatient clinic in June 2013 complaining of chest tightness and shortness of breath for 2 weeks. She denied having hypertension, diabetes mellitus, dyslipidemia, tobacco use, or any family history of atherosclerotic disease. Her vital signs were as follows: blood pressure at 124/80 mmHg, heart rate of 64 beats/minute, respiratory rate of 24 breaths/minute, and body temperature of 36.5°C. Physical examination revealed diastolic murmurs at the cardiac apex, with a grade of 2/6. Electrocardiography was normal, and chest radiography revealed no active lesions in the lungs. Initial laboratory tests were normal, except for mild thrombocytopenia (platelet count of 82,000/µl); hemoglobin was 12.5 g/dl, albumin was 4.9 g/dl, and C-reactive protein was 0.07 mg/dl. Troponin or N-terminal pro-B-type natriuretic peptide tests were not performed initially.

**Figure 1:**
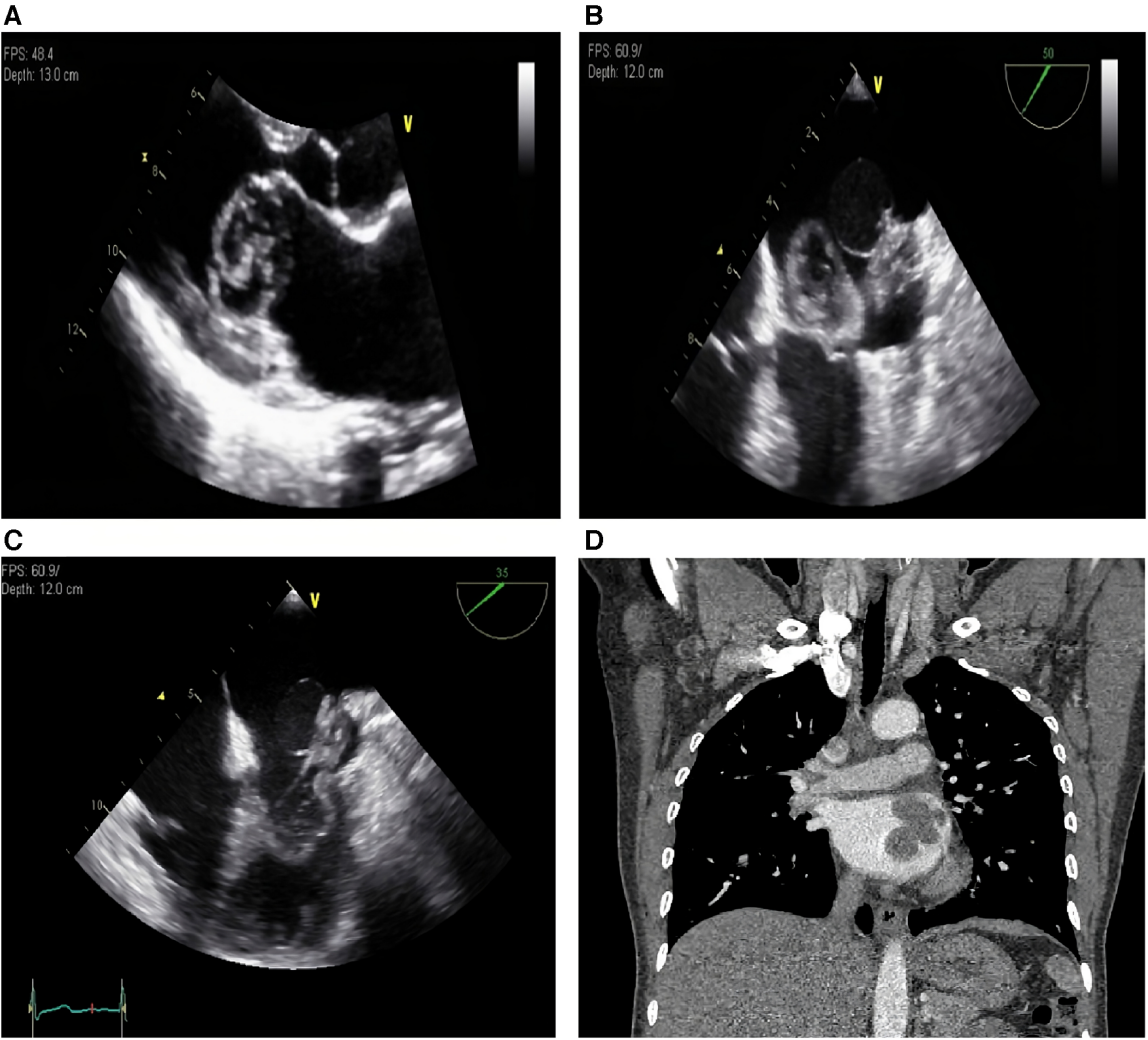
(**A**) Transthoracic echocardiography, parasternal long axis view. A broad-based mass is filling the left atrium and protruding into the LV cavity during diastole. (**B**) Transesophageal echocardiography, systole. The mass which is composed of three different parts are shown. (**C**) Transesophageal echocardiography, diastole. The mixed portion of the mass is protruding into the LV cavity during diastole. (**D**) Thoracoabdominal computed tomography reveals an intracardiac mass occupying the left atrium. LV, left ventricle.

Transthoracic echocardiography (Figure 1A) revealed a broad-based mass filling the left atrium that was attached to its posterior wall near the Q tip. The mass was composed of three different parts: a thin-walled cystic portion (2.3 cm × 2.1 cm), a solid portion with lobulating contours and heterogenic echogenicity (2.8 cm × 2.4 cm), and a thick-walled mixed portion (3.7 cm × 2.9 cm). The mixed portion of the mass protruded into the LV cavity during diastole and caused severe functional mitral stenosis (transmitral mean diastolic pressure gradient, 5 mmHg; mitral valve area, 1.7 cm^2^ by pressure half-time). Transesophageal echocardiography (Figures 1B,C) clearly revealed the heterogeneous features of the mass. Transesophageal echocardiography revealed no evidence of pulmonary vein invasion. Thoracoabdominal computed tomography (CT) (Figure 1D) revealed an intracardiac mass occupying the left atrium without local invasion and no evidence of distant metastasis. Radiography did not reveal any lesions in the skeletal system. The presumptive diagnosis was a benign cardiac tumor, such as an unusual type of left atrial myxoma, and the patient was referred to a cardiac surgeon for surgical removal of the mass.

**Figure 2:**
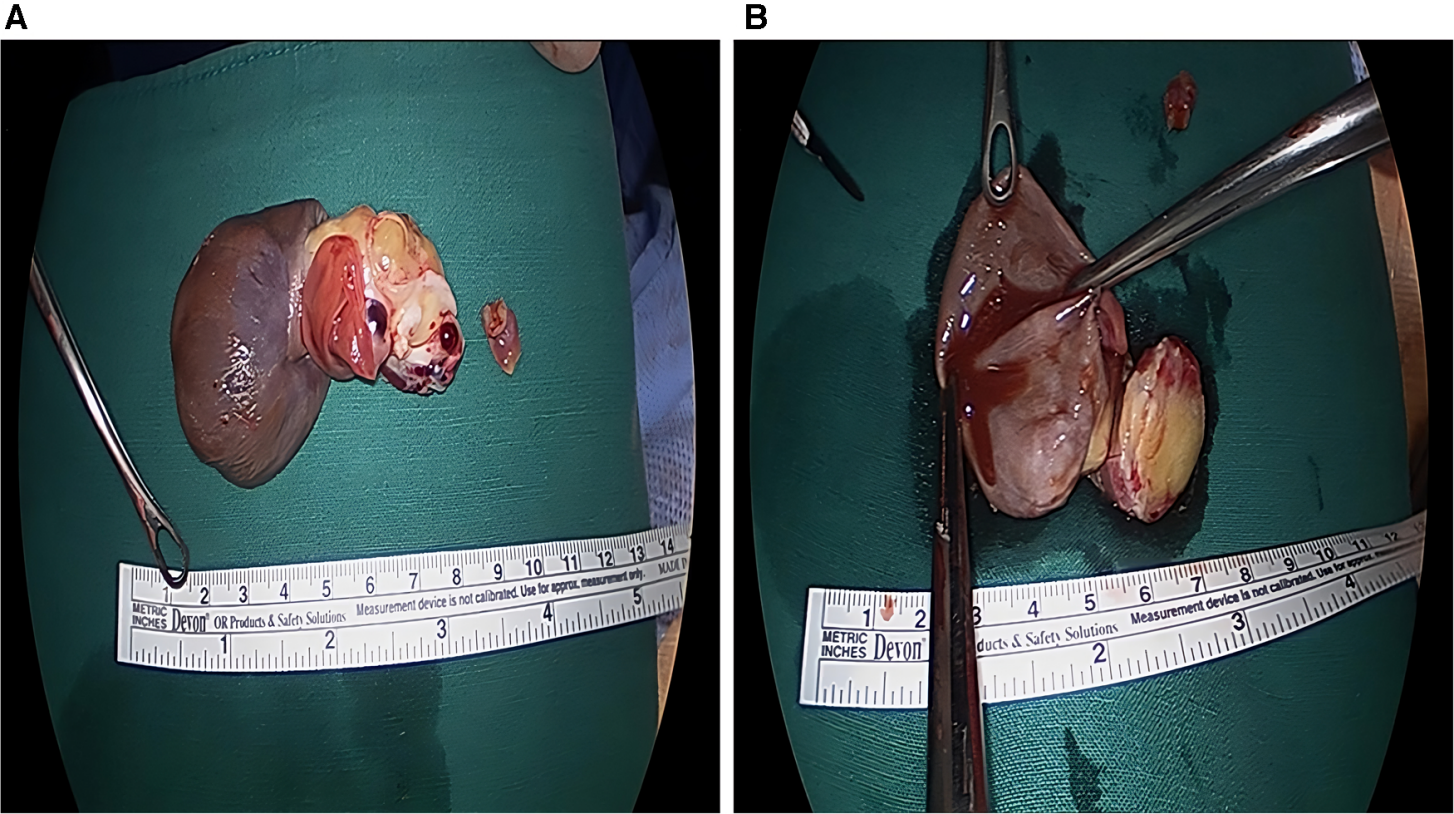
Gross photograph of the mass. (**A,B**) A 7 cm × 5 cm × 4 cm sized huge mass with multilobulated one hard portion and two cystic portion was surgically removed.

**Figure 3:**
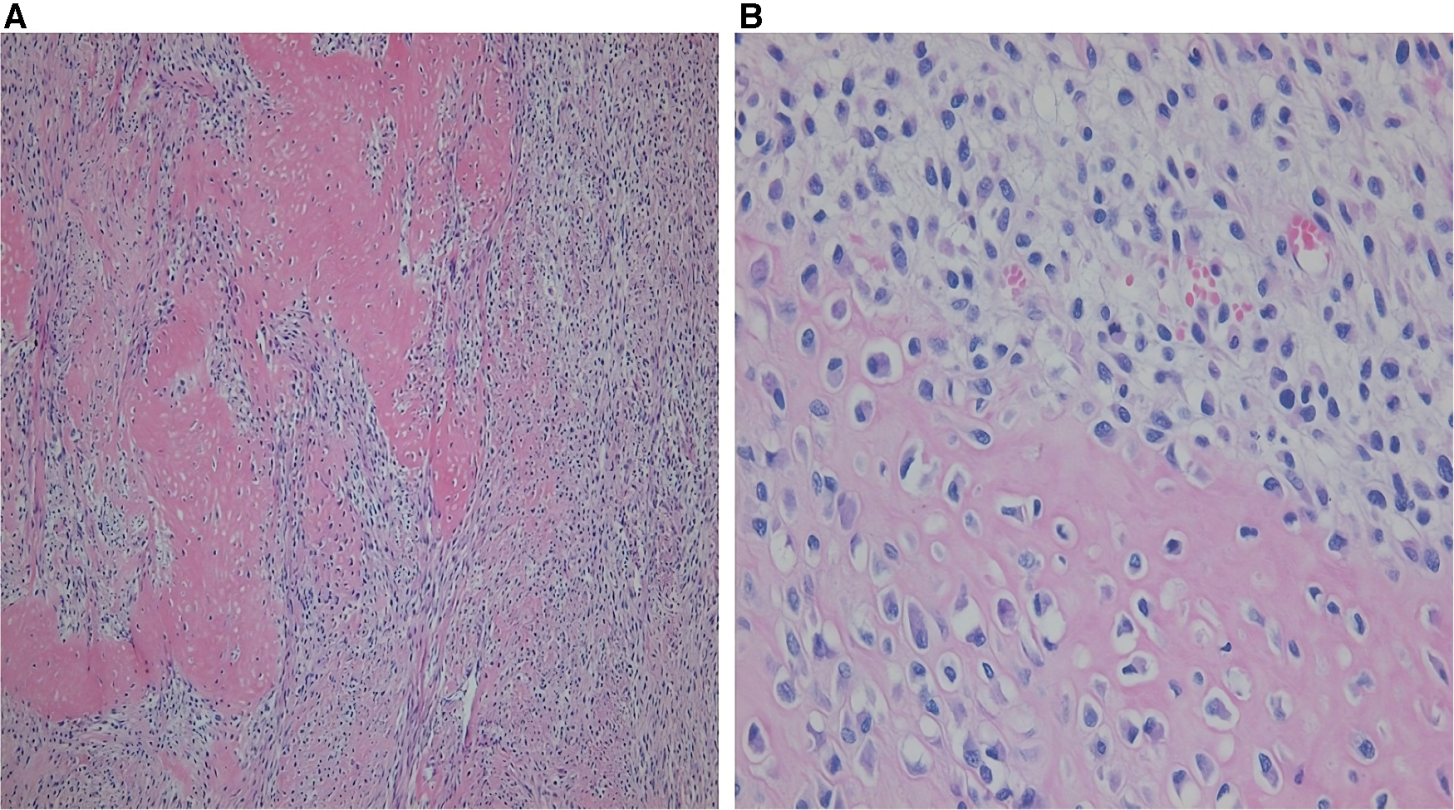
Photomicrograph of the mass. (**A**) The cellular anaplastic area is mixed with cartilage and atypical cells (hematoxylin and eosin staining, X100). (**B**) Atypical pleomorphic osteoblasts are observed, and a cellular anaplastic area with cartilage is present (hematoxylin and eosin staining, X400).

During surgery, a 7 cm × 5 cm × 4 cm mass was observed, which had a hard part originating from the nearby left atrial appendage and two cystic parts (Figures 2A,B). Complete excision of the mass and partial endocardiectomy were performed under cardiopulmonary bypass guidance. On pathological examination, the tumor consisted of proliferating pleomorphic spindle-shaped cells, suggesting a sarcoma, and massive osteoid and chondroid materials were produced by the tumor cells (Figures 3A,B). High mitotic activity (14 per 10 high-power fields) and moderate cytological atypia were detected with scattered hemorrhagic and necrotic foci. The tumor cells invaded the resection margins. Immunohistochemistry revealed positive results for CD68, S-100, and smooth muscle actin. Finally, the tumor was diagnosed as chondroblastic primary cardiac osteosarcoma.

On follow-up echocardiogram performed 1 week after surgery, no cardiac tumors were observed, and no other abnormalities were detected. The patient recovered after surgery and was discharged 2 weeks later.

She received six cycles of systemic chemotherapy with doxorubicin at a dose of 25 mg/m^2^ and cisplatin at a dose of 75 mg/m^2^. During 2 years of follow up, the patient remained healthy with no evidence of tumor recurrence on echocardiography or CT scans. The patient did not receive any further follow-up or treatment until returning to the hospital 10 years later for regular health checkups. Although echocardiography and CT were not performed at that time, the absence of specific symptoms or abnormalities on physical examination suggested that the primary osteosarcoma had resolved.

## 3. Discussion

Primary malignant cardiac tumors are a rare clinical condition representing 15%–20% of all primary cardiac tumors. Primary cardiac osteosarcomas are particularly rare, account for 3%–9% of cardiac sarcomas (6). While extraskeletal osteosarcoma typically occurs in the fifth to seventh decades with a higher incidence in males, primary cardiac osteosarcoma is diagnosed at a slightly younger age (mean age 43.6 years) and does not exhibit gender predominance (7). The specific prevalence of primary cardiac osteosarcoma based on gender or race has not yet been definitively determined, given its rarity in the population. No common genetic lesions or pathway alteration have been confirmed in osteosarcoma, but what is commonly observed is significant aneuploidy and some evidence of massive disruption in the control of chromosomal structure (8).

With the advancement of cardiac imaging techniques and improved accessibility, the diagnosis of cardiac osteosarcoma, which was previously detected mainly through postmortem examinations, has become more straightforward. Cardiac imaging modalities, including echocardiography, cardiac CT, cardiac magnetic resonance imaging (CMR) and nuclear imaging, play complementary roles in evaluating cardiac masses (9). Echocardiography, a widely available and non-invasive imaging technique, provides precise information about the size, shape, location, mobility and hemodynamic impact of cardiac tumors (9–11). Moreover, contrast echocardiography using contrast agents offers supplementary insights into morphology and perfusion of tumors (9–12). Transesophageal echocardiography, particularly when utilizing three-dimensional imaging techniques, affords a comprehensive anatomical assessment of the mass and its adjacent tissues (9–11). Cardiac CT with its high spatial resolution and soft tissue discrimination can clearly demonstrate the relationship between the mass and surrounding structures, allowing for the assessment of local invasion (9, 11). CMR provides sufficient spatial and temporal resolution, allowing for multi-planar reconstruction (9, 11). It offers tissue characterization through inherent soft-tissue contrast, enhanced by various tissue-weighted sequences and the use of gadolinium-based contrast agents (9, 11). CMR effectively distinguishes between benign and malignant cardiac masses, with tumor size, signal intensity heterogeneity, local infiltration, and enhanced initial perfusion serving as reliable indicators of malignancy (9, 13, 14). In addition, ^18^F fluorodeoxyglucose positron emission tomography (FDG-PET) provides valuable information regarding the metabolic activity of cardiac tumors, enabling differentiation between benign and malignant tumors and detecting distant metastasis (9, 15, 16). In this case, CMR and PET were not performed due to the limited accessibility to advanced imaging techniques at the time of the patient's diagnosis in 2013. If these imaging modalities had been conducted prior to the surgery, they could have raised suspicions of malignancy and allowed for a more meticulous preparation for radical excision.

Because primary cardiac osteosarcomas mostly originate in the left atrium (6, 7, 11, 17), they may be misdiagnosed as cardiac myxomas, which are the most prevalent tumors at this site (7, 9). Cardiac myxomas are characterized by a distinguishable stalk and homogenous echogenicity on echocardiography, whereas cardiac osteosarcomas usually present as broad-based masses with heterogeneous echogenicity and may occasionally exhibit internal calcification (6, 10). They typically grow rapidly and often infiltrate the surrounding tissues. Cardiac osteosarcomas are frequently identified as low-attenuation masses with occasional calcifications on computed tomography (18).

However, differentiating cardiac osteosarcoma based solely on imaging findings is difficult, and histopathological diagnosis is essential. In the case of intracardiac tumors, obtaining tissue samples can be challenging because of their location or mobility, and there is a high risk of complications associated with biopsy, such as bleeding, thromboembolic events, and damage to the surrounding tissues. Therefore, in many cases, surgical treatment is performed simultaneously with histological examination. During surgery, a frozen biopsy can be performed to provide a preliminary diagnosis, followed by surgical resection to ensure negative resection margins as much as possible. Whole-body CT or PET-CT should be performed after surgery to evaluate potential metastasis to other organs (19).

The 5-year overall and disease-free survival rates for primary cardiac osteosarcoma are 33.5% and 6.3%, respectively (7, 8, 20), indicating poor prognosis. Radical tumor excision is widely regarded as the foremost treatment modality, and the completeness of tumor resection has a significant impact on prognosis (6, 21, 22). However, complete surgical resection can be challenging because cardiac osteosarcomas are highly invasive and frequently infiltrate adjacent tissues (6, 10, 16). The precise incidence of local recurrence and distant metastasis of cardiac osteosarcoma has not yet been established. However, given the high rate of local recurrence and distant metastasis of osteosarcoma, adjuvant chemotherapy is another important treatment strategy, even in non-metastatic cases (7, 21–23). While the role of adjuvant chemotherapy in primary cardiac sarcomas remains inconclusive, previous studies have consistently shown that postoperative chemotherapy significantly improves survival (7, 22, 24). First-line chemotherapy for osteosarcoma is currently a combination of methotrexate, cisplatin, and adriamycin, which is the standard chemotherapy regimen for skeletal osteosarcomas (25, 26). However, the principles of adjuvant chemotherapy for primary cardiac sarcomas have not yet been established.

Osteosarcomas are rapidly progressing tumors with a high tendency to recur and metastasize, rendering early diagnosis and treatment crucial. Therefore, if osteosarcoma is diagnosed, it is important to determine the presence of metastasis to other organs and promptly perform surgery and chemotherapy. Even with successful treatment, local recurrence and metastasis can frequently occur; therefore, adequate surveillance is necessary.

## 4. Conclusion

We report a case of primary cardiac osteosarcoma, an extremely rare type of primary malignant cardiac tumor, and discuss its characteristic features and treatment methods. Although the patient did not undergo sufficient surveillance, she survived well for up to 10 years. However, if another patient presents with a similar diagnosis, it is important to consider additional tests such as CMR and PET-CT, as well as treatments including radical excision and adjuvant chemotherapy, based on the findings of this report.

## Data Availability

The original contributions presented in the study are included in the article, further inquiries can be directed to the corresponding author.
